# The Danish comorbidity in liver transplant recipients study (DACOLT): a non-interventional prospective observational cohort study

**DOI:** 10.1186/s12876-021-01733-5

**Published:** 2021-04-01

**Authors:** Magda Teresa Thomsen, Julie Høgh, Andreas Dehlbæk Knudsen, Anne Marie Reimer Jensen, Marco Gelpi, Gerda E. Villadsen, Rozeta Abazi, Peter Holland-Fischer, Lars Køber, Otto Clemmesen, Paul Suno Krohn, Jens Hillingsø, Tina Vilsbøll, Tor Biering-Sørensen, Klaus Fuglsang Kofoed, Børge Grønne Nordestgaard, Allan Rasmussen, Susanne Dam Nielsen

**Affiliations:** 1 Viro-Immunology Research Unit, Department of Infectious Diseases 8632, Copenhagen University Hospital, Blegdamsvej 9B, 2100 Copenhagen, Denmark; 2 Department of Hepatology and Gastroenterology, Aarhus University Hospital, Århus, Denmark; 3 Department of Gastroenterology and Hepatology, Odense University Hospital, Odense, Denmark; 4 Department of Medical Gastroenterology, Aalborg University Hospital, Aalborg, Denmark; 5 Department of Cardiology, Rigshospitalet, University of Copenhagen, Copenhagen, Denmark; 6 Department of Gastroenterology and Hepatology, Rigshospitalet, University of Copenhagen, Copenhagen, Denmark; 7 Department of Gastro-Surgery/Liver Transplantation, Rigshospitalet, University of Copenhagen, Copenhagen, Denmark; 8 Steno Diabetes Center Copenhagen, Gentofte Hospital, University of Copenhagen, Copenhagen, Denmark; 9 Department of Cardiology, Herlev-Gentofte Hospital, Copenhagen University Hospital, Copenhagen, Denmark; 10 The Copenhagen General Population Study, Department of Clinical Biochemistry, Herlev Gentofte Hospital, Copenhagen University Hospital Herlev, Herlev, Denmark

**Keywords:** Liver transplantation, Comorbidity, Cardiovascular diseases, Respiratory diseases, Metabolic diseases, Renal diseases

## Abstract

**Background:**

Liver transplantation is the only curative treatment for patients with end-stage liver disease. Short-term survival has improved due to improved surgical techniques and greater efficacy of immunosuppressive drugs. However, long-term survival has not improved to the same extent as the short-term survival, and the 10-year survival after liver transplantation is 60%. In addition to liver- and transplant-related causes, comorbidities such as cardiovascular, pulmonary, renal, and metabolic diseases have emerged as leading causes of morbidity and mortality in liver transplant recipients. The objective of this study is to assess the burden of comorbidities and identify both liver- and transplant-related risk factors as well as traditional risk factors that contribute to the pathogenesis of comorbidity in liver transplant recipients.

**Methods/design:**

The Danish Comorbidity in Liver Transplant Recipients (DACOLT) study is an observational, longitudinal study. We aim to include all adult liver transplant recipients in Denmark (n = approx. 600). Participants will be matched by sex and age to controls from the Copenhagen General Population Study (CGPS) and the Copenhagen City Heart Study (CCHS). Physical and biological measures including blood pressure, ankle–brachial index, spirometry, exhaled nitric oxide, electrocardiogram, transthoracic echocardiography, computed tomography (CT) angiography of the heart, unenhanced CT of chest and abdomen and blood samples will be collected using uniform protocols in participants in DACOLT, CGPS, and CCHS. Blood samples will be collected and stored in a research biobank. Follow-up examinations at regular intervals up to 10 years of follow-up are planned.

**Discussion:**

There is no international consensus standard for optimal clinical care or monitoring of liver transplant recipients. This study will determine prevalence, incidence and risk factors for comorbidity in liver transplant recipients and may be used to provide evidence for guidelines on management, treatment and screening and thereby contribute to improvement of the long-term survival.

*Trial registration* ClinicalTrials.gov: NCT04777032; date of registration: March 02, 2021.

## Background

Liver transplantation is the only curative treatment for patients with end-stage liver disease. Improved surgical techniques and greater efficacy of immunosuppressive drugs to prevent rejection have improved the short-term survival of liver transplant recipients. According to the European Liver Transplant Registry the current 1-year survival rate in liver transplant recipients is 86% [1]. However, long-term survival has not improved to the same extent with 5- and 10-year survival rates of 74% and 60%, respectively [1], and efforts to improve the long-term survival are needed.

In addition to liver- and transplant related causes, cardiovascular, pulmonary, kidney, and metabolic diseases contribute to the increased morbidity and mortality in liver transplant recipients [2–4]. Further knowledge of prevalence, incidence and risk factors for development of comorbidity is needed to identify high-risk individuals, to develop to targeted interventions, and to improve survival.

Cardiovascular diseases (CVD) have emerged as a leading cause of morbidity and mortality in liver transplant recipients [5–7], but the magnitude is not well explored, and a systematic review described a highly variable incidence of 1–41% for CVD [8]. Few studies have included controls, but one study indicated a higher incidence of ECG abnormalities in liver transplant patients than in the background population [9]. However, whether liver transplantation is an independent risk factor for CVD is poorly elucidated, and large, prospective cohort studies are needed to provide further insight into the risk factors and mechanisms driving CVD in liver transplant recipients.

Pulmonary and respiratory diseases, including pulmonary hypertension and hepatopulmonary syndrome are frequently seen in liver transplant candidates prior to transplantation [10]. In the acute post-operative phase after liver transplantation, pulmonary complications are frequent and both infectious and non-infectious pulmonary diseases contribute to morbidity and mortality in the acute phase [11]. The extent to which pulmonary diseases contribute to morbidity beyond the acute phase and the impact on long-term survival is unknown.

Studies have shown that mortality related to renal diseases increases over time post-transplantation [2]. Thus, renal insufficiency may be an important risk factor for late post-transplant mortality in liver transplant recipients. The aetiology leading to post-transplant renal failure is multifactorial and include use of immunosuppressive medication, metabolic syndrome, diabetes, circulating immunocomplexes, and factors that contribute to hepatorenal syndrome [12]. Despite the use of renal-sparing immunosuppression protocols, renal insufficiency remains a concern [2].

Metabolic syndrome, obesity and diabetes are highly prevalent in liver transplant recipients [13]. In addition, the use of certain immunosuppressive medication may induce dyslipidaemia [14]. Non-alcoholic fatty liver disease (NAFLD) is a common liver disease and is considered the liver manifestation of the metabolic syndrome [15]. Patients with NAFLD have an increased risk of cardiovascular diseases, kidney diseases and liver fibrosis [16, 17]. Although these are common comorbidities with potential impact on quality of life, morbidity and mortality, there is limited knowledge about metabolic diseases in liver transplant recipients, and studies to determine the incidence and whether liver transplantation is an independent risk factor for metabolic syndrome are needed.

## Study objectives

The overall objective of this study is to determine the burden of comorbidities in liver transplant recipients and to examine whether this differs from the background population. Furthermore, we want to identify both liver- and transplant-related risk factors as well as traditional risk factors that contribute to the pathogenesis of comorbidity in liver transplant recipients.

- AIM 1: To determine the prevalence and incidence of cardiovascular, respiratory, renal and metabolic diseases in liver transplant recipients, and whether the prevalence and incidence of these comorbidities are different from that of the background population.
- AIM 2: To identify liver- and transplant-related risk factors and traditional risk factors that contribute to the pathogenesis of cardiovascular, respiratory, renal and metabolic diseases in liver transplanted recipients and study potential interactions between these.
- AIM 3: To describe the kinetics of cytokines, chemokines, immune cells, proteomics and metabolomics after liver transplantation and identify biomarkers that can identify liver transplant recipients at high risk of developing cardiovascular, respiratory, renal, and metabolic diseases.

## Methods/design

The DACOLT study is a non-interventional, observational, longitudinal study to be initiated in March 2021. The study aims to include all liver transplant recipients living in Denmark (n = 580) as well as all patients who will be liver transplanted from 01.03.2021 until 01.03.2033 (estimated n = 600). Controls will be included from the Copenhagen General Population Study (CGPS) and the Copenhagen City Heart Study (CCHS).

Liver transplant recipients and controls will have uniformly measured clinical data and information regarding risk factors. Clinically measured primary outcomes include (1) coronary CT angiography, cardiac structure and function determined by echocardiography and cardiac CT, and electrophysiological abnormalities determined by electrocardiogram (ECG), (2) dynamic lung function indices measured by spirometry, (3) estimated glomerular filtration rates, (4) diabetes and dyslipidaemia. Several other outcomes are measured. Table 1 provides a complete overview of all clinical and physical measurements, Table 2 of biochemical measures, and Table 3 an overview of the content of the questionnaires used in the study.

**Table 1:** Clinical measurements and collection of biomaterials

Measurements	Description
Anthropometry	Height: Stadiometer without shoes Weight: Scale (Soehnle, Nassau, Germany) without shoes or heavy clothing Waist and hip circumference: Measuring tape (SECA, Birmingham, UK)
Ankle–brachial-index	Doppler (Sonotrax Basic A, Edan, San Diego, CA, U.S.)
Blood pressure	IntelliVue MP5SC (Philips, Amsterdam, Netherlands)
Electrocardiogram	Cardiosoft V6.73 (GE Healthcare, Buckinghamshire, UK)
Spirometry	EasyOne® ultrasonic spirometer (ndd Medical, Zürich, Switzerland)
eNO	NIOX VERO® (Aerocrine AB, Solna, Sweden)
CT scans	CT angiography, CACS, CT chest, CT upper abdomen. 320-detector MSCT (Canon Aquilion ONE, PRISM, Canon Medical Systems, Otawara, Japan)
Transthoracic echocardiography	Vivid 9 Ultrasound System (GE Healthcare, Horten Norway) M-mode, colour tissue doppler, conventional spectral doppler, 2D speckle tracking, 3-dimensional echocardiography
Biobank	PBMC, plasma, serum

*CACS* coronary artery calcium score, *CT* computed tomography, *MSCT* multi-slice computed tomography, *PBMC* peripheral blood mononuclear cell

**Table 2:** Hematological and biochemical measurements

Measurements	Description
Hematology	Hemoglobin, hematocrit, leucocytes, differential count, thrombocytes, red cell dimensions, complement C3
Electrolytes and iron metabolism	Sodium, potassium, calcium, iron, magnesium, ferritin, transferrin, chloride
Liver/ organ related	Aspartate aminotransferase, alanine aminotransferase, alkaline phosphatase, bilirubin, gamma glutamyltransferase, albumin, antitrypsin, amylase, creatinine kinase, lactate dehydrogenase
Coagulation	Activated partial thromboplastin time, coagulation factor II + VII + X, D-dimer, fibrinogen
Lipids	Total cholesterol, remnant cholesterol, HDL cholesterol, LDL-cholesterol, triglycerides, adiponectin, lipoprotein, apolipoprotein A, B, and E
Metabolic	Non-fasting glucose, fasting-glucose, non-fasting insulin, pro-insulin, C-peptide, fasting-insulin, HbA1c, ethanol, hydrogen carbonate
Renal function	Creatinine, urea, uric acid, eGFR
Thyroid function	Thyroid stimulating hormone, free T3/T4
Immunology	Immunoglobulin A and E, rheumatoid factor IgM
Inflammation	High sensitivity C-reactive protein, immunoglobulin A and B, rheumatoid factor (IgM)

**Table 3:** Overview and examples of variables recording for questionnaires

Variable	Examples
Large questionnaire regarding lifestyle and health	
Smoking	Current, previous and passive smoking. Cumulative pack-years, type, filters, e-cigarettes
Alcohol and drug use	Current and previous alcohol intake, intravenous drug use, cannabis
Environmental exposure	Occupational dusts, organic- and inorganic gasses, vapors, fumes
Demographics	Ethnicity, nationality, marital status, children
Socioeconomic status	Level of education, household income
Work	Employment, income, night-work
Diet	Meat intake, vegetable intake
Daily living	Physical activity, social support, sleeping, stress, sun exposure
Medication use	Prescription and doses
Gender, ethnicity	Ethnicity, place of birth
Perinatal/childhood events	Birthweight, delivery at term, breast feeding, respiratory tract infections
Family history of diseases	Acute myocardial infarction, stroke, asthma, COPD, hypertension, diabetes, cancer, depression
Questions for females	Pregnancies, menarche- and menopause age, abortions
Self-reported disease	Asthma, COPD, allergies, diabetes
Symptoms	Angina, dyspnea, cough, wheeze, sputum, reflux
Fracture Risk Assessment Tool (FRAX®)	
Risk factors for fractures	Previous fracture, parent fractured hip, glucocorticoid use, rheumatoid arthritis
Major Depression Inventory (MDI)	
Self-report mood questionnaire	Lack of energy, feeling restless, appetite changes, feeling less self-confident

*COPD* chronic obstructive pulmonary disease

### Follow-up examinations

**Fig. 1:**
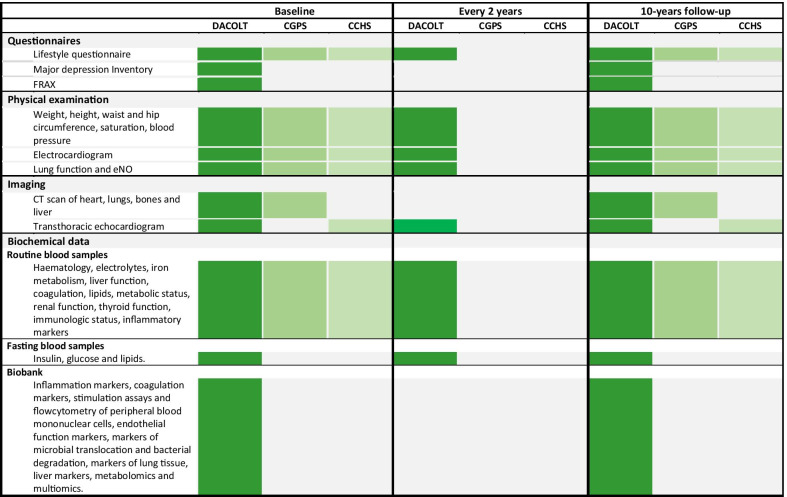
Overview of data collection in the DACOLT study, the Copenhagen General Population Study (CGPS) and the Copenhagen City Heart Study (CCHS). *CT* computed tomography, *FRAX®* Fracture Risk Assessment Tool

The participants in the DACOLT study will be examined at inclusion and after 10 years with extensive questionnaires, eNO test, computed tomography (CT) scans and transthoracic echocardiography (TTE) as well as physical measurements, ECG, spirometry and blood samples. Furthermore, every second year, participants will be invited to participate in a less extensive examination including physical measurements, TTE, ECG, spirometry, blood samples and a smaller questionnaire (Fig. 1).

### Participants

#### DACOLT participants and eligibility criteria

All liver transplant recipient in Denmark aged 18–100 years will be eligible for inclusion in the DACOLT study. Inclusion requires the individual to be able to understand the study information in either Danish or English and to be able to provide an informed consent. Contraindications to the various measures performed in the study (i.e., renal impairment and contraindications to a contrast enhanced CT) do not exclude participants from other parts of the study.

#### Controls from the Copenhagen General Population Study and the Copenhagen City Heart Study

CGPS is an ongoing observational population study with more than 110,000 participants from the greater Copenhagen area. All residents in the greater Copenhagen area > 40 years and 25% of 20–40 years old are invited to participate in the study and in follow-up examinations every decade. A random sample of 10,000 participants aged ≥ 40 years had a contrast enhanced CT of the chest including CT angiography of the heart performed. Of these, 6500 had a contrast enhanced CT of the abdomen.

CCHS includes a random population sample included from the greater Copenhagen area. Health surveys have been repeated 5 times between 1976 and 2015. Almost 4500 participants were randomly selected for echocardiography in 2013–2015.

### Physical and clinical measurements

All data are collected using uniform protocols in the CGPS, CCHS and the DACOLT study (Fig. 1). A physical exam including anthropometrics, pulse, peripheral arterial saturation is performed by trained clinical staff.

#### Blood pressure and ankle–brachial index

Blood pressure is measured electronically on both the right and left arm with the participant in a relaxed seated position using IntelliVue MP5SC (Philips, Amsterdam, Netherlands). Ankle–brachial-index (ABI) is measured in a supine position in both extremities using a Doppler meter (Sonotrax Basic A 294534, Edan, San Diego, CA, US) by determining the systolic pressure of the posterior tibial artery or alternately the pressure of dorsalis pedis artery.

#### ECG

ECG is measured using a 12-lead ECG (Cardiosoft V6.73 GE Healthcare, Buckinghamshire, UK). The ECGs are stored electronically.

#### Spirometry and nitric oxide in exhaled breath

Forced expiratory volume in the first second (FEV1) and forced vital capacity (FVC) are measured by spirometry using an EasyOne World Spirometer (ndd Medical, Zurich, Switzerland) with one-use spirettes (ndd Medical). Spirometry is performed in accordance with American Thoracic Society/European Respiratory guidelines [18]. In participants where the FEV_1_/FVC < 0.7 a repeat spirometry is performed 15 min after bronchodilation with inhalation of 400 µg salbutamol (Ventoline Diskus, Glaxo Smoth Kline, Middlesex, UK). Nitric oxide in exhaled breath (eNO) is measured using NIOX VERO (Aerocrine AB, Solna, Sweden).

### Collection of biomaterials

At time of inclusion blood samples will be collected for routine biochemical analyses (Table 2). A total of 84 ml blood will be collected for a research biobank, and peripheral blood mononuclear cells (PBMC), serum and plasma will be stored for analyses of inflammation markers (chemokines, cytokines, soluble surface markers), coagulation markers (standard and functional platelet aggregation test), simulation assays and flowcytometry of PBMC, endothelial function markers (asymmetric dimethylarginine (ADMA), syndecan-1, thrombomodulin and sE-selectin), markers of microbial translocation and bacterial degradation (sCD14, lipopolysaccharide, trimethylamine N-oxide), markers of lung tissue (alpha-1-antitrypsine, endothelin-1), liver markers (hyaluronic acid and fibrosis markers) and metabolomics and multiomics.

At all follow-up visits, routine blood samples will be drawn and analysed.

### Questionnaires

All participants in DACOLT will complete an extensive questionnaire regarding lifestyle, health, respiratory symptoms and medication, identical to the questionnaire developed and used in the CGPS and CCHS (Table 3). Data based on this questionnaire has been published on several occasions [19–23]. Furthermore, DACOLT participants will also complete a separate questionnaire regarding depression symptoms based on Major Depression Inventory [24] (International Classification of Diseases (ICD)-10) and complete the Fracture Risk Assessment Tool (FRAX®) [25] which is an online algorithm with 12 questions to assess the risk of hip fracture. All these questionnaires will be completed at time of inclusion and at the 10 year follow up. A less extensive questionnaire will be completed at 2-, 4-, 6- and 8-year follow-up.

### CT scans

CT scans will be performed with a 320-detector MSCT (Canon Aquilion ONE, PRISM, Canon Medical Systems, Otawara, Japan) at Rigshospitalet, University of Copenhagen, using identical protocols for DACOLT and GCPS participants. First, in backrest and while holding the inspiration, a high-resolution CT will be performed including all lung parenchyma. Afterwards, CT scan of the heart, liver and abdominal fat will be performed. At last, an intravenous contrast agent (Visipaque 78 ml, 320 mg/ml) will be infused with a flowrate of 5 ml/s followed by a saline chaser and contrast enhanced thoracic and abdominal CT angiography will be performed. The complete CT scan protocol includes several scans performed in the following sequence: Unenhanced CACS, scan of the upper abdomen, abdominal single slice acquisition for measurements of visceral adipose tissue, low-dose chest CT and a contrast enhanced coronary angiography with iodixanol (Visipaque®, GE Healthcare, Brøndby, Denmark). Oral β_1_-antagonist (Metocar, STADA Nordic, Herlev, Denmark) will be administered to patients with a heart rate > 60 bpm and systolic blood pressure > 110 mmHg unless contraindicated. Participants with moderate to severe COPD/asthma will receive Ivabradin 15 mg (Procoralan, Servier, Fredriksberg, Denmark) instead of a β_1_-antagonist. Immediately before CT angiography is performed, one dose of 0.4 mg oral spray nitroglycerin (Nitrolingual, Pohl Boskamp, Hohenlockstedt, Germany) is administered unless contraindicated. The maximum allowed radiation dose for the complete sequence is 8 mSv, and CT will not be performed in patients where the expected radiation dose exceeds 8 mSv.

### Transthoracic echocardiography

Echocardiography will be performed using a pre-specified comprehensive protocol using the Vivid 9 Ultrasound System (GE Healthcare, Horten, Norway). Participants will be examined in the left lateral decubitus position with conventional echocardiography; including M-mode, and colour tissue doppler (TDI), 2D speckle tracking for deformation analyses (strain) as well as 3-dimensional echocardiography. Echocardiograms will be stored in a GE Healthcare image vault, and images will be analysed offline with commercially available post-processing software.

### Other data sources

For DACOLT participants, liver- and transplantation-related variables will be retrieved from patient records. In Denmark, all liver transplant recipients are routinely examined annually with liver biopsies, magnetic resonance imaging (MRI), including MR elastography, and routine blood samples. Furthermore, information on vital status, medicine use and discharge diagnosis can be retrieved from National Danish registries. The Civil Registration System (CRS) contains information on vital status, date of emigration and death of every resident in Denmark [26]. The Danish National Patient Register (NPR) contains information on all hospital admissions to Danish hospitals including discharge diagnoses according to the ICD-10 from 1994 [27]. From the NPR, data on admissions due to cardiovascular, lung, bone, metabolic, and liver diseases will be retrieved. Information regarding purchase and use of medication related to these diseases will be retrieved from the registry of Medicinal Product Statistics that contains information on medicine since 1994, including all prescription and non-prescription drug sales from pharmacies, and all deliveries from general practitioners and hospitals [28].

### Sample size

The current study includes several endpoints, and the necessary sample size varies accordingly.

Previous data from the CGPS have shown coronary atherosclerosis in 50% of the background population [29]. In the following we assume that 90% of the invited liver transplant recipients are included in the study.

Assuming coronary atherosclerosis in 50% of the CGPS participants [29], an alpha of 5%, 540 liver transplant recipients and 5400 controls, we will, with a power (1-β) of 0.90, be able to show a prevalence ratio of 1.15 for atherosclerosis. Assuming ECG changes in 11% of CGPS participants [30], an alpha of 5%, 540 liver transplant recipients and 5,400 controls, we will with a power (1-β) of 0.82 be able to show a prevalence ratio of 1.4 to have ECG abnormalities. In the Danish general population, 1 unit (%) decrease in absolute global longitudinal strain (GLS) is associated with 10% increased risk of heart disease [31]. Assuming an alpha of 1%, a standard deviation of 3.5%, 540 liver transplant recipients and 2,160 controls, we will be able to show a difference of 1% GLS with a power (1-β) of 0.99.

### Planned statistical analyses

Descriptive statistical analyses will be used to assess demographics, risk factors, and physical measurements. Groups will be compared using t-test or Mann–Whitney U test for continuous data and χ2 tests or Fisher’s test for categorical data. The large cohort of controls permits each liver transplant recipients to be matched on age and sex with between 5 and 20 controls. In cross-sectional studies the prevalence of the respective diseases among liver transplant recipients will be compared to the prevalence among the control group using logistic regression adjusted for traditional and/or presumed risk factors. Likewise, numeric variables will be compared using linear regression adjusted for traditional and/or presumed risk factors. For prospective studies, Cox regression, Kaplan–Meier curves, log-rank test, and mixed effect models will be used. In general, missing data is expected to be low and multiple imputations are not likely to be required. Characteristics for loss to follow-up will be assessed.

### Ethical considerations

All participants provide oral and written informed consent before study inclusion. The DACOLT study (H-20052199), CGPS (H-KF-01-144/01) and CCHS (HEH-2015-045) have obtained approval from the Ethics Committee of the Capital Region, Denmark.

CT scans expose participants to a radiation dose of maximum eight millisieverts (mSv). Although low, this radiation dose may cause an increase in lifetime risk of radiation induced cancer. Before giving consent to the CT scan, participants have received both written and oral information about the radiation dose and cancer risk. The CT scans will be reviewed and described by a radiologist. The participants will, if they provide consent, receive information if there are signs of clinically significant disease. Participants will give a specific consent to the storage of biological samples in a research biobank. All adverse events will be recorded.

## Discussion

The 10-year survival after liver transplantation is 60%. In addition to liver- and transplant-related causes, comorbidities such as cardiovascular, pulmonary, renal, and metabolic diseases are leading causes of morbidity and mortality in liver transplant recipients. The primary goal of this study is to identify the burden and risk factors of post-transplant comorbidities and thereby contribute to knowledge of comorbidities, in order to target interventions and to help improve long-term survival. The primary outcome measures are defined within the different disease categories i.e., cardiovascular-, respiratory-, renal- and metabolic comorbidities. Furthermore, the study will establish a research biobank for studies assessing the pathogenesis of comorbidities in liver transplant recipients.

The burden of comorbidity in liver transplant recipients is not well described, and to our best knowledge there is no international consensus standard for optimal clinical care, screening programs or monitoring of liver transplant recipients [32–34].

This study was designed to assess post-transplant comorbidities in liver transplant recipients. Accordingly, the main strengths are the multimodal assessment of risk factors associated with post-transplantation comorbidities and the use of a large control cohort with uniform data collection. Additionally, the use of Danish registry data will allow linkage between data from the DACOLT cohort and cause and date of death, hospital discharge and prescription medicine.

There are also limitations to this study. Limitations include selection bias such as healthy volunteer bias, as liver transplant recipients with more severe illness or disability might be less likely to participate. Furthermore, there might be a geographical selection bias, as some of the inclusion (CT scan) takes place in the capital Copenhagen, which may result in people living outside Copenhagen being less likely to participate in this part of the study. Controls from the CGPS and CCHS are included at random, whereas DACOLT participants are included from the outpatient clinics when they are there for routine controls. Exact reason for non-participation will not be recorded, but the number of non-participations will be registered. Since the study is a prospective cohort study there might also be selection bias in participants lost to follow up. As the study collects information on early life events through self-reported questionnaires, there may be some risk of recall bias, albeit this risk is also present for the control groups.

In conclusion, despite improvements of the short-term survival after liver transplant recipients, the long-term survival remains poor, and efforts to improve the long-term survival are needed. This study has the potential to determine prevalence, incidence and risk factors for development of comorbidity in liver transplant recipients and may be used to develop guidelines on screening, monitoring and long-term treatment of liver transplant recipients and thereby improve survival. Use of data will be confined to the study group, but potential collaborators or request for data can be submitted to dacolt.rigshospitalet@regionh.dk.

## Data Availability

Use of data will be confined to the study group, but potential collaborators or request for data can be submitted at dacolt.rigshospitalet@regionh.dk.
